# Quercetin suppresses lung cancer growth by targeting Aurora B kinase

**DOI:** 10.1002/cam4.891

**Published:** 2016-10-05

**Authors:** Zhu Xingyu, Ma Peijie, Peng Dan, Wang Youg, Wang Daojun, Chen Xinzheng, Zhang Xijun, Song Yangrong

**Affiliations:** ^1^ Department of RespiratoryThe Second Affiliated Hospital to Shanxi College of Traditional Chinese MedicineXianyang712000China; ^2^ Department of PharmacyBaoji Central HospitalBaoji721008China; ^3^ Department of Pulmonary MedicineAffiliated Hospital of Yan'an UniversityYan'an716000China; ^4^ Department of Chest SurgeryAnkang Central Hospital of Shanxi ProvinceAnkangShanxi725000China; ^5^ Department of NephrologicalBaoji city chinese medicine hospitalBaoji721001China; ^6^ Department of Thoracic SurgeryChang ‘an Hospital in Xi'anXi'an Shaanxi710016China; ^7^ Department of Chest SurgeryTumor Hospital of Shannxi ProvinceXi'anShannxi710061China

**Keywords:** aurora B, inhibitor, lung cancer cells, quercetin

## Abstract

aurora B kinase is highly expressed in several cancer cells and promotes tumorigenesis and progression, and therefore, it is an important target for drug to treat tumors. Quercetin was identified to be an antitumor agent. Herein, we report for the first time that quercetin inhibited aurora B activities by directly binding with aurora B in vitro and in vivo. Ex vivo studies showed that quercetin inhibited aurora B activities in JB6 Cl41 cells and A549 lung cancer cells. Moreover, knockdown of aurora B in A549 cells decreased their sensitivities to quercetin. In vivo study demonstrated that injection of quercetin in A549 tumor‐bearing mice effectively suppressed cancer growth. The phosphorylation of histone 3 in tumor tissues was also decreased after quercetin treatment. In short, quercetin can suppress growth of lung cancer cells as an aurora B inhibitor both in vitro and in vivo.

## Introduction

Currently, tumor drug development has changed from traditional cytotoxic drugs to the agents that target the special molecules 1. For example, imatinib, a BCR‐Abl tyrosine kinase inhibitor, was a typical representative 2. There are mainly four categories to target treatment for cancer: kinase inhibitors, chaperone inhibitors, histone deacetylase inhibitors, and inhibitors of protein–protein interactions. Herein, aurora B kinase inhibitors were studied.

Aurora B is a serine–threonine kinase, a member of Aurora family. Aurora B also is a protein that functions in the attachment of the mitotic spindle to the centromere. In cancerous cells, overexpression of aurora B causes unequal distribution of genetic information, creating aneuploidy cells, a hallmark of cancer 3. Aurora B was confirmed to be highly expressed in several malignancies and played an important role in cancer development and progression 4, 5, 6. So, aurora B might be an excellent drug target for cancer chemotherapy.

Currently, several aurora B inhibitors have some side‐effects, such as antiproliferation toxicity on the bone marrow, neutropenia, alopecia, anemia, dry skin, and so on 7, 8, 9. Therefore, it is urgent and beneficial to find aurora B inhibitors, which are highly effective in suppressing aurora B activity with low toxicity. In this study, we employed the structure‐based microscale thermophoresis (MST) method to screen the compound from plant and found that quercetin can block aurora B directly and inhibit its activity.

## Material and Methods

### Reagents and antibodies

Mouse epidermal JB6 Cl41 cells and human lung cancer cells A549, H1975, and H441 were purchased from ATCC, Virginia, USA. Commercial quercetin was obtained from Weikeqi Bioscience, China. Antibodies against phospho‐histone H3 (S10), aurora B, histone H3 were obtained from Cell Signaling Technology, Danvers, MA. The active aurora B protein for the kinase assay was purchased from Millipore, USA. Signal Chem. Antibodies to detect *β*‐actin were purchased from Santa Cruz Biotechnology, CA.

### Cell culture

JB6 Cl41 was cultured at 37°C in a 5% CO_2_ incubator in MEM medium containing 5% fetal bovine serum (FBS). A549, H1985, and H441 cells were cultured at 37°C in a 10% CO_2_ incubator in RPMI‐1640 medium containing 10% FBS, respectively. After starvation in serum‐free medium for 24 h, cells were treated with quercetin at the indicated concentration and time. Cells were then stimulated with EGF (20 ng/mL) for 30 min.

### MTS assay

To estimate cytotoxicity, JB6 Cl41, A549, H1975, and H441 cells were seeded (8 × 10^3^ cells per well) in 96‐well plates and cultured overnight. The attached cells were then fed with fresh medium and treated with various concentration of quercetin. After culturing for various times, the cytotoxicity of quercetin was measured using an MTS (3‐(4,5‐dimethylthiazol‐2‐yl)‐5‐(3‐carboxymethoxyphenyl)‐2H‐tetrazdium) assay kit (Promega, Madison, WI). The absorbance was read at 490 nm according to the instructions. All the experiments were performed in triplicate, and the mean absorbance values were calculated.

### Anchorage‐independent transformation assay

Mouse epidermal JB6 Cl41 cells (8 × 10^3^/mL) or were exposed to EGF (20 ng/mL) with or without quercetin (0–100 *μ*mol/L) in 1 mL of 0.33% Basal Media Eagle (BME) agar containing 10% FBS, 2 mmol/L L‐glutamine, and 25 *μ*g/mL gentamicin. The cultures were maintained at 37°C, in a 5% CO_2_ incubator for 10 days and colonies were counted using a microscope Motic AE 20 (China) and the Motic Image Plus computer program (Media Cybernetics, Silver Spring, MD). Human lung cancer cells were treated as described above instead of exposing to EGF.

### Microscale thermophoresis

Aurora B protein was labeled with the Monolith NT^™^ Protein Labeling Kit RED (Cat#L001) (NanoTemper Technologies GmbH, Germany) according to the supplied labeling protocol. The aurora B protein were diluted in a 20 mmol/L HEPES (pH 7.4) and 0.05 (v/v) % Tween‐20. The quercetin stock was dissolved in ddH_2_O in a concentration of 5 mmol/L. We used 5 mmol/L quercetin as the highest concentration for the serial dilution. After 10 min incubation at room temperature, the samples were loaded into MonolithTM standard‐treated capillaries and the thermophoresis was measured at 25°C after 30 min incubation on a Monolith NT.115 instrument (NanoTemper Technologies, München, Germany). Laser power was set to 20% or 40% using 30 sec on‐time. The LED power was set to 100%. The dissociation constant Kd values were fitted using the NTAnalysis software (NanoTemper Technologies, München, Germany) 10.

### Western blot

The harvested cells were lysed with lysis buffer and protein concentrations of cells were determined by the Bradford method. Lysate protein (10 g) was subjected to 10% sodium dodecyl sulfate‐polyacrylamide gel electrophoresis (SDS‐PAGE) and transferred onto polyvinylidene fluoride membrane (PVDF), which were blocked with 5% nonfat milk and then incubated with a specific primary antibody at 4°C for overnight. Proteins were visualized using a chemiluminescence detection kit after hybridization with a horseradish peroxidase‐conjugated secondary antibody. All the experiments were performed in triplicate, and band density was quantified using the Image J 12.0 software (National Institutes of Health, Bethesda, USA).

### In vitro binding assay

A549 cell lysates (1 mg) were incubated with the quercetin, or quercetin‐Sepharose 4B beads in the reaction buffer (50 mmol/L Tris [pH 7.5], 5 mmol/L ethylenediaminetetraacetic acid, 150 mmol/L NaCl, 1 mmol/L dithiothreitol, 0.01% Nonidet P‐40, 2 *μ*g/mL bovine serum albumin, 0.02 mmol/L phenylmethylsulfonyl fluoride, and 1 *μ*g/mL protease inhibitor mixture). After gentle rocking overnight at 4°C, the beads were washed five times and proteins were analyzed by western blot. All the experiments were performed in triplicate.

### In vitro kinase assay

Inactive histone H3 proteins were used as the substrate for an in vitro kinase assay with active aurora B. Active aurora B was incubated with quercetin (10, 20, and 50 *μ*mol/L) and 100 *μ*mol/L ATP in 1 × kinase buffer (25 mmol/L Tris‐HCl pH 7.5, 5 mmol/L beta‐glycerophosphate, 2 mmol/L dithiothreitol, 0.1 mmol/L Na_3_VO_4_, 10 mmol/L MgCl_2_) at 32°C for 90 min. Reactions were stopped and proteins were detected by western blot. All the experiments were performed in triplicate.

### Xenograft mouse model

Athymic nude mice [NIH Swiss nude, 6–9‐week‐old] were obtained from Beijing HFK Bioscience CO., LTD (Beijing, China). The animals were maintained at the Laboratory Animal Center, The Fourth Military Medical University (China). The animals were divided into two groups, vehicle group and quercetin‐treated group (*n* = 10 of each group). A549 colorectal cancer cells (4 × 10^6^/0.1 mL) were injected subcutaneously into the right flank of each mouse. Treatment was started when the tumors reached a mean volume of 100 mm^3^. Quercetin or vehicle was administered to mice three times a week for 21 days by intraperitoneal (i.p.) injection. Tumor volumes and body weights were measured. The mice were monitored until tumors reached 1 cm^3^ total volume, at which time the mice were killed and the tumors were extracted. The tumors were embedded in a paraffin block and subjected to immunohistochemistry (IHC) or hematoxylin and eosin (H&E) staining. All animal experiments were performed following the protocols approved by the Laboratory Animal Center of the Fourth Military Medical University.

### Statistical analysis

All quantitative data were expressed as mean values ± standard deviation, and significant differences were determined by Student's test or by one‐way ANOVA. A probability value of *P* < 0.05 was used as the criterion for statistical significance.

## Results

### Quercetin binds with aurora B by MST and in vitro binding assay

**Figure 1:**
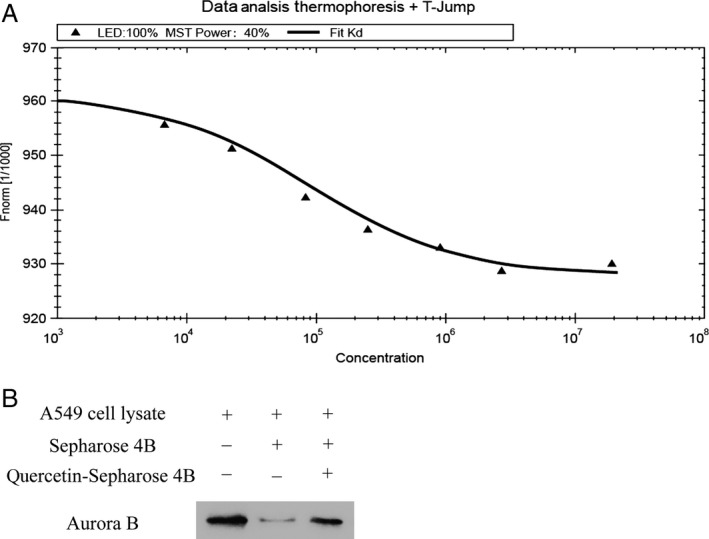
Quercetin can bind with aurora B. (A) Measurement of affinity between quercetin and aurora B by MST in standard treated capillaries, and the resulting binding curve are shown. From the resulting binding curve, Kd of 25.6 ± 3.5 was calculated. (B) Quercetin binds directly with aurora B in vitro. Sepharose 4B was used for binding and pull‐down assay. Lane 1 is input control (aurora B protein standard); Lane 2 is the negative control, indicating no binding between aurora B and beads alone; and Lane 3 indicates that aurora B binds with quercetin‐Sepharose 4B beads. MST, microscale thermophoresis.

We detected the binding affinity between the antitumor compounds and aurora B using MST method. This technology can quantify protein and small molecule interactions with high sensitivity and low sample cost by detecting fluorescent changes of molecules during thermophoresis. The result showed the lowest equilibrium dissociation constant (Kd) of 25.6 ± 3.5 *μ*mol/L (Fig. 1A, Table 1), which meant the strongest binding between the quercetin and aurora B.

**Table 1:** Binding affinity and inhibitory activities of screening hits

	ICM docking mfscore^a^	Dissociation constant with aurora B	Inhibitory activities against A549 cells
Compound	(kcal/mol)	Kd^b^ (*μ*mol/L)	IC50 (*μ*mol/L)
Quercetin	−156	25.6 ± 3.5	176.5
Curcumin	−134	346 ± 8.9	n.i^c^
Naringin	−76.32	564 ± 13.6	n.i^c^
Resveratrol	−74.54	263 ± 25.8	n.i^c^

a Docking score/interaction potential of compounds with Aurora B (kcal/mol).

b The Kd value is automatically calculated by the curve fitting, and presented as means±SD.

c n.i. is no inhibition detected in the experiments.

To validate the veracity of the MST method, we employed the in vitro beads binding assay to assess the binding between quercetin and aurora B in A549 cells which has high expression of aurora B. No obvious band representing aurora B was observed in beads without quercetin group, whereas a strong band was seen in quercetin‐conjugated beads group (Fig. 1B).

### Quercetin suppresses EGF‐induced anchorage‐independent growth of JB6 Cl41 cells

**Figure 2:**
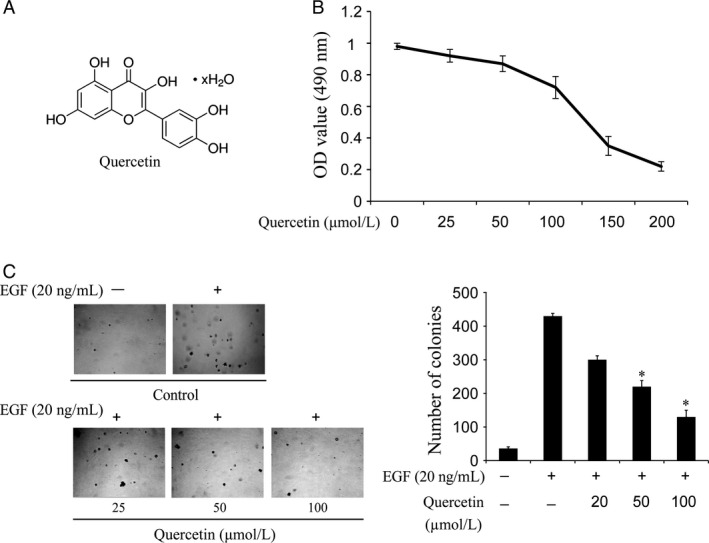
Quercetin inhibits EGF‐induced anchorage‐independent growth of JB6 Cl41 cells. (A) The chemical structure of quercetin. (B) Cytotoxic effects of quercetin on JB6 Cl41 cells. An MTS assay was used after treatment of cells with quercetin for 24 h. (C) Quercetin inhibits EGF‐induced anchorage‐independent growth of JB6 Cl41 cells. JB6 Cl41 cells (8 × 10^3^) were exposed to EGF (20 ng/mL) and treated with quercetin (0–50 *μ*mol/L) in 1 mL of 0.3% Basal Medium Eagle (BME) agar containing 10% fetal bovine serum (FBS), 2 mmol/L L‐glutamine, and 25 *μ*mol/L gentamicin. The cell colonies were scored using a microscope Motic AE 20 (China). Data are shown as mean ± standard deviation from triplicate experiments. The asterisk (*) indicates a significant inhibition (*P < *0.05) by quercetin in colony formation.

To determine the cytotoxicity of quercetin, different concentrations of the drug were used to treat JB6 Cl41 cells for 24 h. Cytotoxicity was measured by MTS assay and the results indicated that quercetin did not decrease the viability of JB6 Cl41 cells up to 100 *μ*mol/L at 24 h (Fig. 2B). Next, we investigated whether quercetin had an effect on anchorage‐independent growth of JB6 Cl41 cells treated with only epidermal growth factor (EGF). The results indicated that JB6 Cl41 cells treated with different concentration of quercetin formed fewer colonies compared with the control group (Fig. 2C). For example, colony formation was inhibited by more than 30% after treatment with quercetin at a concentration of 50 *μ*mol/L, and almost 60% colonies were inhibited at 100 *μ*mol/L quercetin (Fig. 2C). These results showed that quercetin could attenuate EGF‐induced anchorage‐independent JB6 Cl41 cell growth and was not cytotoxic within a proper concentration range.

### Quercetin inhibits aurora B activity in vitro and ex vivo

**Figure 3:**
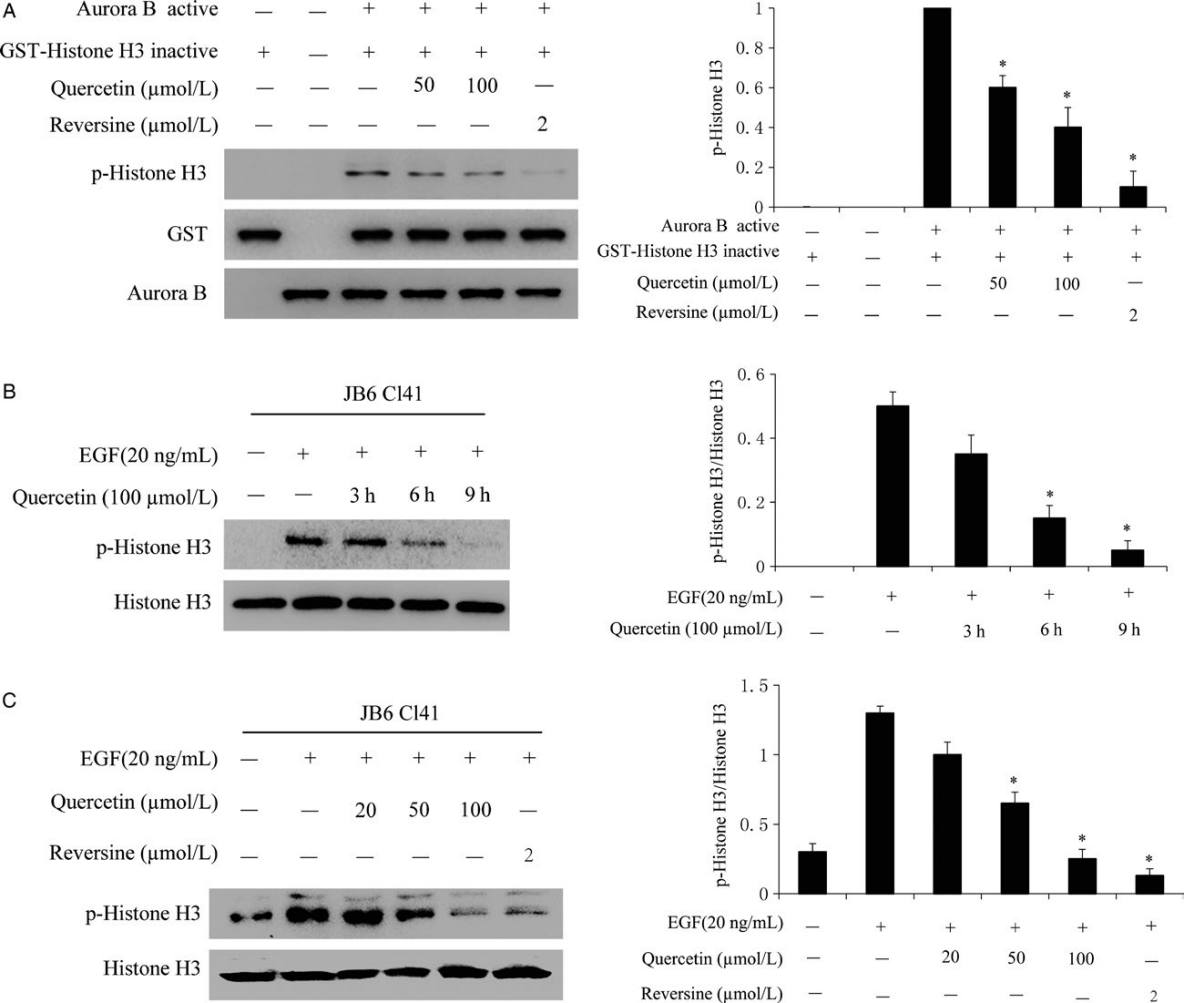
Quercetin suppresses aurora B activity in vitro and ex vivo. (A) Quercetin inhibits aurora B activity in vitro. The inhibitory effect of quercetin on aurora B was determined by an in vitro kinase assay as described in section “Materials and methods”. Data are representative of results from triplicate experiments and the asterisk (*) indicates a significant difference (*P < *0.05) compared to aurora B kinase and H3 substrate group (lane 3). (B) Quercetin inhibits aurora B activity in JB6 Cl41 cells. The cells were starved 24 h and then treated with the presence or absence of 100 *μ*mol/L quercetin for various times as indicated. After the cells were treated with 20 ng/mL EGF for 30 min, histones were extracted from cells, total histone H3 and phosphorylated histone H3 proteins were detected by western blot using specific antibodies. Data are representative of results from triplicate experiments and the asterisk (*) indicates a significant difference (*P < *0.05) compared to EGF group. (C) After starvation in serum‐free medium for 24 h, cells were treated with quercetin at the indicated concentration for 9 h and then stimulated with EGF (20 ng/mL) for 30 min. Data are representative of results from triplicate experiments and the asterisk (*) indicates a significant difference (*P < *0.05) compared to EGF group.

Our previous data showing that quercetin directly binds with aurora B, suggesting that quercetin might inhibit the aurora B activity. To confirm this hypothesis, we performed an in vitro kinase assay with histone H3 as the substrate with active aurora B in the presence of 25, 50, 100 *μ*mol/L of quercetin. The results showed that phosphorylation of histone H3 (Ser10) was substantially attenuated in a dose‐dependent manner after treatment with quercetin (Fig. 3A). Reversine, a novel aurora B inhibitor, was used as a positive control 11. Furthermore, we examined whether quercetin could suppress aurora B activities in JB6 Cl41 cells. Data indicated that phosphorylation of histone H3 (Ser10) was lessened by treatment with quercetin in a time‐dependent (Fig. 3B) and dose‐dependent manner (Fig. 3C).

### Quercetin inhibits anchorage‐independent growth of lung cancer cells

**Figure 4:**
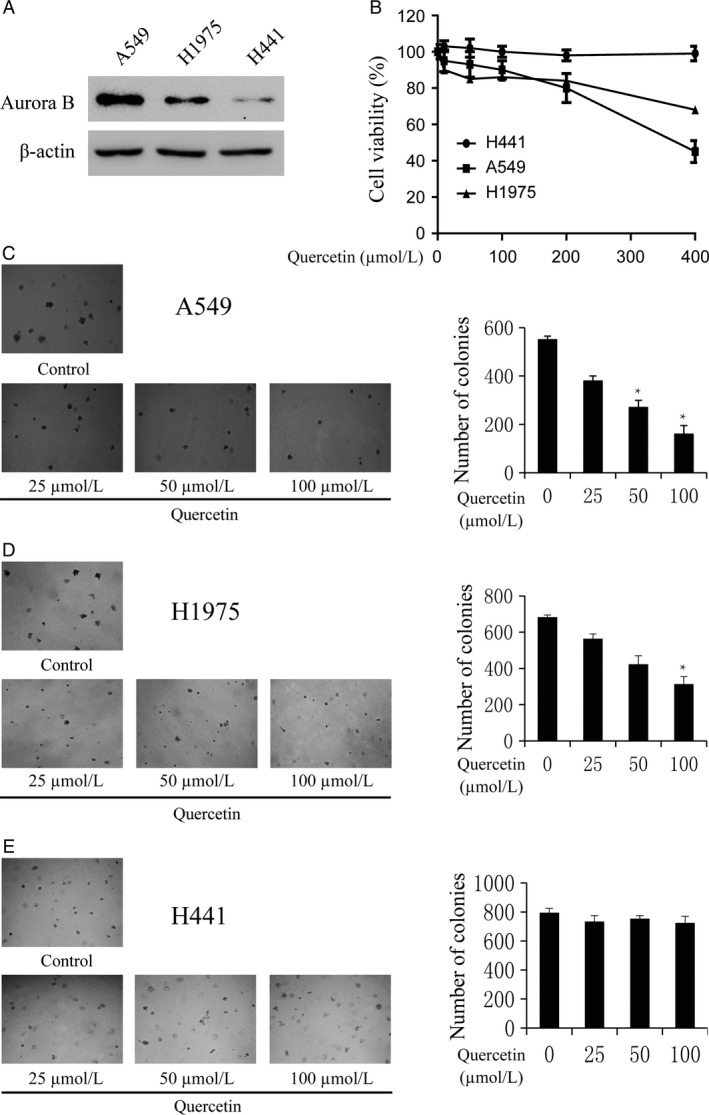
Quercetin inhibits anchorage‐independent growth of colorectal cancer cells. (A) Expression of aurora B in lung cancer cell lines A549, H1975, and H441. (B) Different concentrations of quercetin were used to treat lung cancer cell lines for 48 h, respectively. Cytotoxicity was measured by MTS assay. (C–E) The effect of quercetin on anchorage‐independent growth of lung cancer cell lines with different level of aurora B expression, including A549 cells (C), H1975 cells (D), and H441 cells (E). The cells were treated with quercetin (0–100 *μ*mol/L) for 14 days, and the cell colonies were scored using a microscope Motic AE 20 (China). Data are shown as means ± standard deviation of values from three independent experiments and the asterisk (*) indicates a significant difference (*P < *0.05) compared to control group.

Previous studies revealed that aurora B is highly expressed in human lung cancer 12, 13, 14. We attempted to determine whether quercetin could affect anchorage‐independent growth of lung cancer cells. We used three lung cancer cell lines A549, H1975, and H441 with high, middle, and low expression level of aurora B, respectively (Fig. 4A). Firstly, we determine the cytotoxicity of quercetin by MTS assay. Different concentrations of the drug were used to treat lung cancer cell lines A549, H1975, and H441 for 48 h, respectively. The results indicated that quercetin had different cytotoxicities toward different lung cancer cells. A549 cells with high aurora B expression were more sensitive to quercetin (Fig. 4B). The colony numbers of the cells were counted after culturing for 7 days using different concentrations of quercetin. The results showed that quercetin at 25, 50, and 100 *μ*mol/L inhibited colony formation of A549 cells on 27, 49, and 83%; H1975 cells on 25, 37, and 62%; and H441 on 5, 12, and 24%, respectively, compared with the nontreated cells (Fig. 4C–E). Overall, our results suggested that inhibitory effect of quercetin on colony formation was significant in A549 cells with a high expression level of aurora B.

### Knocking down aurora B in A549 cells decreased the sensitivity of quercetin

**Figure 5:**
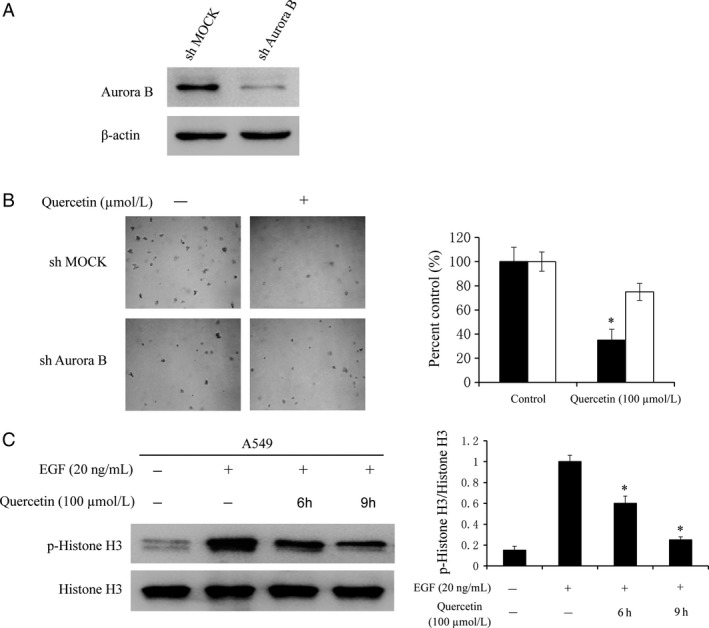
Knocking down aurora B attenuates the inhibitory effect of lung cancer cell growth by quercetin. (A) Efficiency of aurora B shRNA in A549 cells. (B) Anchorage‐independent growth of A549 cells transfected with shMOCK or sh‐aurora B. Data are represented as mean ± standard deviation from triplicate experiments. The asterisks (*) indicate a significant decrease (*P < *0.05) compared to shMOCK cells. (C) Quercetin inhibits aurora B activity in A549 cells. A549 cells were starved in a serum‐free medium for another 24 h. Next, the cells were treated with quercetin (100 *μ*mol/L) for different time, and then treated with EGF (20 ng/mL) for 30 min. The cells were then harvested and the protein levels were determined by western blot. Data are representative of results from triplicate experiments and the asterisks (*) indicate a significant decrease (*P < *0.05) compared to EGF group.

To investigate whether the effects of quercetin are mediated directly through aurora B, first we determined the efficiency of sh‐aurora B, as well as the effect of sh‐aurora B transfection on anchorage‐independent growth. The expression of aurora B was obviously decreased after sh‐aurora B (Fig. 5A). Moreover, quercetin suppressed anchorage‐independent growth in shMock cells but had less effects in sh‐aurora B cells (Fig. 5B). Next, western blot results indicated that the phosphorylation level of histone H3 (Ser10) was substantially decreased with quercetin treatment in a time‐dependent manner (Fig. 5C). The above results showed that aurora B is a direct target for quercetin to suppress lung cancer cells growth.

### Quercetin suppresses tumor growth by inhibiting aurora B activity in vivo

**Figure 6:**
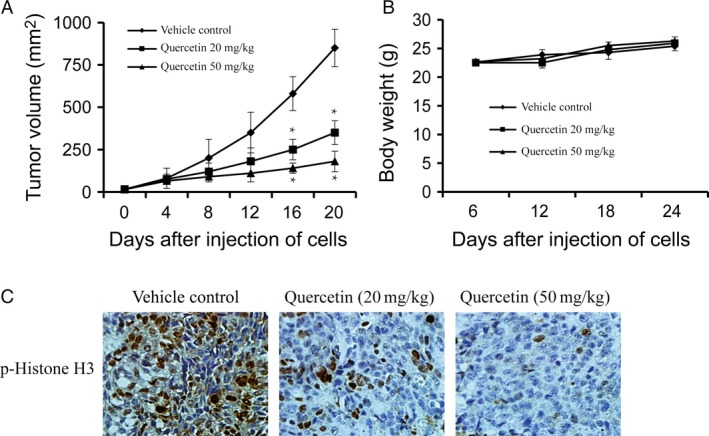
Effect of quercetin on lung cancer growth in an A549 xenograft mouse model. (A) Quercetin significantly suppresses cancer growth in an A549 xenograft mouse model. The average tumor volume of vehicle‐treated control mice (*n* = 8) and quercetin‐treated mice (*n* = 8) plotted over 20 days after tumor cell injection. The asterisk (*) indicates a significant increased tumor size (*P < *0.05). (B) Quercetin has no effect on mouse body weight. Body weights from the treated or untreated groups of mice were measured once a week. (C) Quercetin inhibits the expression of phosphorylated histone H3 in vivo. Immunohistochemistry analysis was used to determine the level of phosphorylated histone H3 in tumor tissues.

To explore the antitumor efficacy of quercetin in xenograft model, we injected subcutaneously into the left flank of 6‐week‐old athymic nude mice using A549 cells. The mice were divided into two matched groups. Vehicle or quercetin treatment was injected when the tumors reached a mean tumor volume of 100 mm^3^. The data indicated that tumors treated with 50 mg/kg quercetin grew significantly more slowly and the size of tumors was smaller compared with the vehicle group (Fig. 6A). However, the weight of mice has no significant change between the vehicle and quercetin‐treated group (Fig. 6B), which indicated that the dose of quercetin used for the experiment had no toxicity to the mice. To further determine whether the antitumor effect of quercetin was associated with its inhibition of aurora B activities, tumor extracts from either group were analyzed immunohistochemically. The results showed that expression level of phosphorylated histone H3 was substantially decreased in the quercetin‐treated group compared with the vehicle group (Fig. 6C). Overall, our results indicated that quercetin suppressed tumor growth by inhibiting aurora B activities in vivo.

## Discussion

Lung cancer is increasing gradually year by year, with an overall 5‐year survival rate of only 15% 15. Despite the potential of special targeted small molecule therapy in advanced lung cancer, almost all patients ultimately develop resistance to tyrosine kinase inhibitor because of EGFR mutation 16, 17, 18. Therefore, it is a good strategy to inhibit other signaling pathway to overcome TKIs resistance of lung cancer. Previous studies showed that lung cancer was treated through inhibiting aurora B signaling pathway. 14, 19, 20.

aurora B, a collection of highly related serine/threonine kinases, is a member of Aurora kinases family. aurora B is a mitosis regulator which plays a critical role in performing accurate chromosomal alignment and segregation. 21. Previous studies showed that inhibition of aurora B kinase resulted in cell‐cycle arrest or even death 22. The protein kinase activity of aurora B gradually increases at S phase and peaks at G2‐M phase in parallel with elevation of their mRNA and protein expression 23, 24, 25. Subsequently, the kinases are degraded by the proteosome upon exit from mitosis through the ubiquitin‐dependent APC/c pathway 26, 27. aurora B is highly expressed in several malignancies, especially in lung cancer. aurora B overexpression was significantly correlated with aneuploidy and poor prognosis in non‐small cell lung cancer 28. As aurora B expression is limited to proliferating cells, inhibitors of aurora B would be expected to show clinical utility distinct from that of antitubulin compounds as well as other antimitotic drugs known to cause a mitotic arrest 29. This suggested that aurora B could be a potential target for chemotherapeutic treatment of lung cancer.

MST screening was performed as a novel way to screen a selective aurora B inhibitor from several potential antitumor natural compound. We identified a natural compound, quercetin, can inhibit the proliferation of lung cancer by blocking aurora B activity in vitro and in vivo.

Nature compound have high efficacy and less toxicity, and gain more and more interests to search for their potent phamaceutical values in chemoprevention and chemotherapy. 30, 31. Quercetin is a flavonoid compounds in many fruits, vegetables, leaves, and grains. It can be used as an ingredient in supplements, beverages, or foods. Quercetin is derived from *quercetum* (oak forest), and has been reported to possess antiaging 32, anticancer 33, antiinflammation 34, antioxidative 35 effects. Previous studies showed that quercetin inhibited lung cancer cells growth and metastasis 36, 37, 38. Quercetin also can enhance chemosensitivity of lung cancer cells 39. Although quercetin is a pleiotropic protein kinase enzymes inhibitor 40, the molecular mechanism of its pharmacological action is still incomplete in lung cancer cells. Herein, we found a new target of quercetin. Quercetin can target aurora B kinase directly and inhibit the proliferation of lung cancer.

In conclusion, we provided evidence showing that quercetin effectively suppressed anchorage‐independent cell growth of lung cancer cells with highly expressed aurora B levels, and suppressed tumor growth of A549 cells by inhibiting aurora B activities in vivo. Overall, our findings offer an alternative therapy or enhance the efficacy of radiotherapy for lung cancer by targeting aurora B with quercetin.

## Conflicts of Interest

The authors have declared that there is no conflict of interest.
